# Fulminant *Yersinia Pestis* Infection in a Known HIV-Positive Patient: A Case Report of Identification in Nigeria 2024

**DOI:** 10.1093/cid/ciaf512

**Published:** 2025-11-20

**Authors:** Vivian Gga Kwaghe, Cyril Erameh, Nankpah Godsave Vongdip, Osahogie Isaac Edeawe, Onyia Justus Ejike, Blessed Okhiria, Lauren P Courtney, Claire A Quiner, Jean H Kim, Adamu Zigwai Ephraim, Jay Osi Samuels, Philippe Chebu, Oladimeji Damilare Matthew, Victoria Orok, Ikponmwosa Odia, Jacqueline Agbukor, Femi Owolagba, Ephraim Ogbaini-Emovon, Walter Mary Odion, Blessing Amierhobhiye Obagho, Richard Fayomade, Emmanuel A Oga

**Affiliations:** University of Abuja Teaching Hospital, Federal Capital Territory, Nigeria; Irrua Specialist Teaching Hospital, Edo, Nigeria; University of Abuja Teaching Hospital, Federal Capital Territory, Nigeria; Irrua Specialist Teaching Hospital, Edo, Nigeria; University of Abuja Teaching Hospital, Federal Capital Territory, Nigeria; University of Abuja Teaching Hospital, Federal Capital Territory, Nigeria; RTI International, Durham, North Carolina, USA; RTI International, Durham, North Carolina, USA; RTI International, Durham, North Carolina, USA; RTI International, Durham, North Carolina, USA; APIN Public Health Initiatives, Federal Capital Territory, Nigeria; APIN Public Health Initiatives, Federal Capital Territory, Nigeria; University of Abuja Teaching Hospital, Federal Capital Territory, Nigeria; University of Abuja Teaching Hospital, Federal Capital Territory, Nigeria; Irrua Specialist Teaching Hospital, Edo, Nigeria; Irrua Specialist Teaching Hospital, Edo, Nigeria; APIN Public Health Initiatives, Federal Capital Territory, Nigeria; Irrua Specialist Teaching Hospital, Edo, Nigeria; Irrua Specialist Teaching Hospital, Edo, Nigeria; Irrua Specialist Teaching Hospital, Edo, Nigeria; APIN Public Health Initiatives, Federal Capital Territory, Nigeria; RTI International, Durham, North Carolina, USA; ClineEpi Partners, Columbia, Maryland, USA

## Abstract

*Yersinia pestis* is one of the most dangerous pathogens in the world. While plague foci exist in Africa, no recent cases have been reported in Nigeria. We describe a fatal case of *Y. pestis* infection in a known HIV-positive patient who presented to a tertiary health facility in Nigeria. This case underscores the need for high clinical suspicion, even in regions considered non-endemic, and highlights the potential severity of co-infection in immunocompromised individuals.

*Yersinia pestis*, a gram-negative, non-motile, non-spore-forming coccobacillus, is the causative agent of plague and has been designated a Category A bioterrorism agent. It is transmitted primarily by fleas (*Xenopsyllacheopis*) from rodent reservoirs and has caused devastating epidemics throughout history. Since 2001, 14 major outbreaks have been reported to the World Health Organization (WHO), mostly from Africa and Asia [1]. No case has been reported in Nigeria in over 30 years.

There are three primary clinical forms of plague: bubonic, septicemic, and pneumonic. Less common manifestations include gastroenteritis, conjunctivitis, pharyngitis, meningitis, and endophthalmitis [2]. Common initial symptoms are high fever, chills, malaise, and headache. Bubonic plague accounts for 70%–90% of cases and is the most common form. Primary septicemic plague involves a fatal systemic infection without buboes [3]. Pneumonic plague occurs when the lungs are involved, either through hematogenous spread or direct inhalation, and is characterized by high fever, cough, and pleuritic chest pain. HIV-1 infection leads to progressive immune system compromise, increasing susceptibility to a wide range of opportunistic infections. While co-infections in HIV-positive individuals are common, to our knowledge, this is the first reported case of co-infection with HIV and *Y. pestis*.

## CASE REPORT

A 44-year-old man with a known history of HIV infection, who had been on highly active antiretroviral therapy (HAART) for 15 years, presented to the University of Abuja Teaching Hospital, Nigeria, with a 5-day history of high-grade fever, headache, and vomiting, followed by new-onset seizures. He had defaulted from his antiretroviral therapy four months prior to the onset of the illness.

There was no history of cough, chest pain, rash, neck stiffness, or bleeding from any orifice. He had no history of recent travel, known contact with a sick person or animal, or exposure to someone with similar symptoms. Notably, he resided in a house located directly behind a local abattoir—a potential source of zoonotic pathogens.

On examination at presentation, he was acutely ill, febrile (temperature: 38.0°C), dehydrated, and unconscious with a Glasgow Coma Score of 5/15 (E2, V2, M1). He had pinpoint pupils sluggishly reactive to light, global hypotonia, but no signs of meningeal irritation. Cardiovascular and gastrointestinal examinations were unremarkable. Respiratory rate was elevated at 30 breaths per minute; oxygen saturation was 91% on room air. Auscultation revealed coarse crepitations in both lung fields. Blood pressure was 120/80 mmHg, and pulse rate was 116 bpm.

**Initial Management:**

A presumptive diagnosis of sepsis with possible meningeal involvement, with a differential diagnosis of intracranial space-occupying lesion in an HIV-positive patient, was made. The patient was commenced on:

- Intranasal oxygen (4 L/min).
- Intravenous normal saline (1 L every 8 h).
- Empiric broad-spectrum antibiotics:
  - Ceftriaxone 2 g 12-hourly.
  - Vancomycin 600 mg 8-hourly.
  - Metronidazole 500 mg 8-hourly.
- Intravenous dexamethasone (10 mg 8-hourly).
- Anticonvulsants: levetiracetam and intravenous diazepam (10 mg for breakthrough seizures).

**Laboratory Investigations:**

Key abnormalities included:

- Acute kidney injury: Creatinine 546 µmol/L (Reference range 64–104), Urea 24.8 mmol/L (Reference range 2.1–7.1).
- Metabolic acidosis: Bicarbonate 15 mmol/L (Reference range 20–30).
- Elevated liver enzymes: AST 255 U/L, ALT 86 U/L (Reference range < 40).
- Hypoalbuminemia: Albumin 27 g/L (Reference range 35–52).
- Anemia: Hemoglobin 9.0 g/dL, PCV 26%.
- Thrombocytopenia: Platelet count 86 × 10^9^/L.
- Leukocytosis with neutrophil predominance: WBC 11.7 × 10^9^/L; Neutrophils 80%.

These findings were consistent with severe sepsis, organ dysfunction, and possible disseminated intravascular coagulation (see Table 1).

**Table 1.** Baseline Laboratory Investigation Results

Test	Result	Reference Range	Unit
**Electrolytes, Urea and Creatinine**			
Sodium	146	136–148	mmol/L
Potassium	4.1	3.0–5.0	mmol/L
Chloride	107	98–110	mmol/L
Bicarbonate	15	20–30	mmol/L
Urea	24.8	2.1–7.1	mmol/L
Creatinine	546	64–104 (Male)	µmol/L
**Liver Function Tests**			
Total Bilirubin	14.0	5–21	µmol/L
Direct Bilirubin	6.6	<3.4	µmol/L
Alkaline Phosphatase	140	F < 240/M < 270	U/L
Aspartate Transaminase (AST)	255	<40	U/L
Alanine Transaminase (ALT)	86	<40	U/L
Total Protein	82	66–87	g/L
Albumin	27	35–52	g/L
**Complete Blood Count**			
White Blood Cell Count (WBC)	11.7	4.0–11.0	×10^9^/L
Red Blood Cell Count (RBC)	3.5	4.5–5.5	×10¹²/L
Hemoglobin (Hb)	9.0	11.0–18.0	g/dL
Packed Cell Volume (PCV)	26	35–52	%
Mean Cell Volume (MCV)	75.9	78–94	fL
Mean Cell Hemoglobin (MCH)	25.6	25–33	pg
Mean Cell Hb Conc. (MCHC)	33.7	31–37	g/dL
Platelets	86	100–450	×10^9^/L
**Differential Count**			
Neutrophils (%)	80	40–75	%
Lymphocytes (%)	16	25–45	%
Monocytes (%)	4	2–10	%

### Detection of *Yersinia pestis*

With consent from the family, the patient was enrolled in the *Surveillance of Acute Febrile Illness Aetiology in Nigeria* research study [4]. As part of the study protocol, 3 mL of blood was obtained and analyzed using a real-time polymerase chain reaction (PCR) screening tool via Thermo Fisher's TaqMan® Array Card (TAC), which is a research-use-only tool that screened for 25 bacterial, viral, and protozoal pathogens. The TAC test returned positive for *Y. pestis*.

### Clinical Course and Outcome

Despite aggressive supportive care, the patient's condition continued to deteriorate. He remained unconscious, with persistent respiratory distress, metabolic derangements, and evidence of multi-organ dysfunction. No targeted anti-plague antibiotics (eg, streptomycin, gentamicin, doxycycline, ciprofloxacin) were administered, as *Y. pestis* was detected post-mortem.

He died 48 h after admission.

## DISCUSSION

Plague remains a severe and potentially fatal zoonotic disease caused by *Y. pestis*, which has been responsible for three major pandemics throughout history. The current, third pandemic—termed the “modern plague”—originated in Yunnan province, China, in the late 18th century and spread globally through steamship and rail transport [5]. While plague foci are active in parts of Africa and Asia, countries like the Democratic Republic of Congo, Madagascar, and Peru continue to report the majority of cases. Historically, *Y. pestis* has been documented in Nigeria, most notably during a major outbreak in Lagos between 1924 and 1931, as described by Faleye in *Plague and Trade in Lagos, 1924–1931* [6]; however, there has been no confirmed case in over 30 years.

Our patient, an HIV-positive man who had defaulted from HAART for 4 months, presented with signs of severe systemic illness, including high-grade fever, altered mental status, respiratory distress, and laboratory evidence of multi-organ dysfunction. These manifestations are consistent with the clinical profile of primary septicemic plague, a form of *Y. pestis* infection that progresses rapidly and is often fatal.

Septicemic plague is associated with severe bacteremia in the absence of buboes, and may present with features of septic shock, disseminated intravascular coagulation, and multi-organ failure. This patient's biochemical profile demonstrated acute kidney injury, transaminitis, hypoalbuminemia, and thrombocytopenia, all of which are indicative of systemic inflammatory response and end-organ damage. His severe thrombocytopenia, in the absence of overt bleeding, raises the possibility of internal hemorrhage due to consumptive coagulopathy.

The high leukocyte count with neutrophilic predominance, in conjunction with respiratory findings (coarse crepitations and hypoxia), also raises the likelihood of pneumonic involvement**—**either primary or secondary—although imaging studies were not done. Altered mental status in the context of sepsis and HIV could have been due to metabolic encephalopathy, central nervous system infection, or HIV-associated neurocognitive disorder.

HIV infection is well recognized for increasing susceptibility to opportunistic infections due to progressive immunosuppression. In Nigeria, co-infections with tuberculosis, herpes viruses, and hepatitis viruses are commonly reported among HIV-positive individuals [7–10]. However, to the best of our knowledge, co-infection with HIV and *Y. pestis* has not been previously reported in the literature. This case may represent the first such documented instance globally.

The virulence of *Y. pestis* is attributable to a unique set of factors, particularly Yersinia outer-membrane proteins (Yops), which inhibit phagocytosis, downregulate pro-inflammatory cytokines, and disrupt host immune cell function [11, 12]. These immune evasion strategies are particularly devastating in patients with underlying immunosuppression, such as untreated or poorly controlled HIV.

While plague is highly treatable if recognized early, the prognosis worsens dramatically with delays in diagnosis or the absence of targeted antimicrobial therapy [13]. The patient in this case received broad-spectrum empiric antibiotics (ceftriaxone, vancomycin, metronidazole), but none of the first-line agents effective against *Y. pestis*—such as streptomycin, gentamicin, doxycycline, or ciprofloxacin—were administered. Unfortunately, the pathogen was detected post-mortem as part of an ongoing febrile illness surveillance study.

The source of infection remains unclear, but the patient's residence behind a local abattoir suggests possible rodent exposure, a known transmission pathway in plague ecology. Abattoirs can attract rodents and fleas, and poor environmental sanitation may facilitate transmission in urban settings.

This case illustrates the challenges of diagnosing rare or reemerging infections in non-endemic settings, particularly in immunocompromised patients. It underscores the critical need for rapid diagnostic capacity, awareness among clinicians, and robust public health surveillance systems to detect and respond to emerging infectious threats.

## CONCLUSION

This report underscores the importance of maintaining a high index of suspicion for *Y. pestis* infection, even in regions where plague has not been recently reported. To the best of our knowledge, this is the first documented case of HIV and *Y. pestis* co-infection. It highlights the need for enhanced surveillance, improved diagnostic capacity, and prompt initiation of appropriate therapy for reemerging infectious diseases.
